# Conjunctival Lymphangiogenesis Was Associated with the Degree of Aggression in Substantial Recurrent Pterygia

**DOI:** 10.1155/2016/1592514

**Published:** 2016-01-28

**Authors:** Wei Zhao, Tao Wang, Juan Deng, Lei Zhong, Weilan Huang, Shiqi Ling

**Affiliations:** ^1^Department of Ophthalmology, Guangzhou Women and Children's Medical Center, Guangzhou Medical University, Guangzhou 510623, China; ^2^Department of Ophthalmology, The Third Affiliated Hospital, Sun Yat-Sen University, Guangzhou 510630, China

## Abstract

*Objective*. To examine conjunctival lymphatic vessels and to analyze the relationship between lymphangiogenesis and aggressive recurrent pterygia.* Methods*. Tissues from 60 excised recurrent (including 19 of Grade 1, 28 of Grade 2, and 13 of Grade 3) pterygia were used in the study. Tissues from 9 nasal epibulbar conjunctivae segments were used as controls. Pterygium slices from each patient were immunostained with LYVE-1 monoclonal antibodies to identify lymphatic microvessels in order to calculate the lymphovascular area (LVA), the lymphatic microvessel density (LMD), and the lymphovascular luminal diameter (LVL). The relationship between lymphangiogenesis (LVA, LMD, and LVL) and pterygium aggression (width, extension, and area) was clarified.* Results*. Few LYVE-1 positive lymphatic vessels were found in the normal epibulbar conjunctiva segments. Lymphatic vessels were slightly increased in Grades 1 and 2 and were dramatically increased in Grade 3 recurrent pterygia. The LMD was correlated with the pterygium area in Grade 1 and 2 pterygia. In Grade 3, both LVA and LMD were significantly correlated with the pterygium area.* Conclusions*. Lymphangiogenesis was associated with the degree of aggression in recurrent pterygia, particularly in substantial Grade 3 recurrent pterygia.

## 1. Introduction

Pterygium is one of the most common ocular surface diseases. Millions of people worldwide, particularly in specific regions such as Tibet and Hainan provinces, have pterygia. A pterygium is a benign condition characterized by a wing-shaped fibrovascular conjunctival growth that has advanced onto the cornea. This tissue is harmless unless it has overgrown on the corneal region, at which point it threatens patients' vision and may require surgical excision [1]. The main challenge in surgically managing pterygia is the issue of recurrence. To prevent recurrence, various adjunctive treatments, including chemotherapy, radiotherapy, and surgical transplants, have been employed [2]. However, according to many published studies, recurrence rates remain high following pterygium, ranging between 7.5% and 44.4% and undermining the current operative methods and adjuvant treatments [3–5]. Therefore, studies on recurrent pterygium have received more and more attentions in recent days.

The roles of inflammation and fibrovascular proliferation have been highlighted in the pathogenesis of recurrent pterygia. Angiogenesis has been demonstrated during pterygium formation and progression [6, 7]. Vascular endothelial growth factor receptor 2 (VEGFR-2) expression may have predictive value in pterygium recurrence [8]. Vascular endothelial growth factor (VEGF) blockade using specific antibodies such as bevacizumab is able to suppress neovascularization and pterygium recurrence [9–11]. According to recent studies (human and animals), a parallel relationship exists between angiogenesis and lymphangiogenesis after corneal (and/or conjunctival) inflammation, injuries, and transplantation [12–15]. The following questions remain. (1) Do lymphatic vessels develop in pterygium progression and recurrence? (2) Does conjunctival lymphangiogenesis, similar to angiogenesis, play a role in the formation of recurrent pterygium?

We recently found an increasing lymphatic microvessel density (LMD) in primary pterygia. Compared with blood vessels, the LMD was greater in Grade 2 and 3 pterygia. The LMD in Grade 2 and 3 pterygia was approximately double and triple, respectively, that of Grade 1 pterygia. The increasing rate of the blood microvessel density (BVD) in Grade 2 pterygia was less than 20% of that in Grade 1. Thus, the outgrowth of lymphatic vessels (lymphangiogenesis) may play an important role in substantial pterygia [16]. In a three-year follow-up study of 96 primary pterygium patients, the pterygium recurrence time (RT) was calculated, and the relationship of the RT with the LMD and/or BVD was analyzed. The RT was not correlated with the BVD but was negatively correlated with the LMD. Increased lymphatic vessels suggested increased pterygia recurrence [17].

The aims of this study were to provide further evidence that conjunctival lymphangiogenesis develops in recurrent pterygia and to examine the relationship between lymphangiogenesis and the progression of pterygium recurrence. The findings from this study may broaden our understanding of the mechanisms of pterygium development.

## 2. Materials and Methods

### 2.1. Subjects

This observational, comparative study was conducted at the Department of Ophthalmology of the Third Affiliated Hospital of Sun Yat-Sen University from January 2008 to June 2015. This investigation was conducted in accordance with the Declaration of Helsinki tenets for research involving human subjects and was approved by the Institutional Review Board of the Third Affiliated Hospital of Sun Yat-Sen University. A total of 60 recurrent pterygium patients (25 males and 35 females) with a mean age of 54.9 years (range: 35–71 years) and a mean RT of 22.5 months (range: 2.5–56.5 months) were enrolled in the study. The pterygium apex was invading the cornea by a minimum of 1 mm. Clinical evaluations were performed by the same ophthalmologist, as previously described [18]. Pterygia were preoperatively graded based on vascularity, conjunctival congestion and edema, relative fibrovascular lesion thickness, and general eye redness on a scale of 1–3: 1+ (mild), 2+ (moderate), and 3+ (severe). The degree of pterygium aggression, including the horizontal extension from the limbus onto the cornea, and the width of the base at the limbus were measured (in mm) under a slit lamp. The total area was calculated (Table 1). Nine nasal epibulbar conjunctival segments near the limbus, excised from nine age-matched control patients who had strabismus surgery, were used as control tissues. All the patients and controls were informed of the experimental nature of this procedure, and signed consent was obtained.

### 2.2. Immunohistochemistry

Excised conjunctival segments were fixed in 10% neutral formalin for 24 h, embedded in paraffin, serially sectioned to a 4 *μ*m thickness, rehydrated with graded ethanol-water mixtures, and washed with distilled water. Endogeneous peroxidase activity was blocked after incubation in 30 mL/L hydrogen peroxidase for 20 min. Tissue sections were autoclaved at 121°C in 10 mmol/L citrate buffer (pH 6.0, 10 min) for antigen retrieval. Sections were cooled at room temperature for 30 min. Sections were incubated for 3 h with mouse anti-human LYVE-1 monoclonal antibody (R&D Systems, MN) and biotin-marked rabbit anti-mouse immunoglobulin secondary antibody. Streptavidin-biotin complex- (SABC-) peroxidase was used as the immune check system. The slides were visualized for peroxidase activity with diaminobenzidine (DAB) and counterstained with hematoxylin.

### 2.3. Quantification of Immunohistochemical Staining

Slices were viewed using a Zeiss Axioskop microscope, and images were projected to a Sony PVM1440QM video monitor using a Sony CCDIRIS video camera. Digitized images were captured with a Fujix HC-1000 3CCD high resolution color camera. After preliminary scanning of each slice at low power, five areas of high lymphovascular density were imaged at high power (100x) and captured for further analysis using Axiovision 4.7.2 (Carl Zeiss, Jena, Germany).

### 2.4. Lymphovascular Area (LVA) Quantification

Computer images were converted into a threshold raw binary format that highlighted the LYVE-1 stained lymphatic vessels with minimal background. These images were then analyzed using an in-house computer image analysis program that reported the proportion of the area occupied by immunostained lymphatic vessels.

### 2.5. Lymph Microvascular Density (LMD) Quantification

The number of stained lymph microvessels on the computer images was manually counted. Each vessel was marked after being counted to avoid repetition. Vessel counts per field were represented as vessels per *μ*m^2^.

### 2.6. Lymphovascular Luminal Diameter (LVL) Quantification

The maximum luminal diameter of a stained microvessel with a clear lumen was manually measured using the computer images. Each vessel was marked after being measured to avoid repetition.

### 2.7. Statistical Analysis

Significant differences among groups were determined using one-way analysis of variance (ANOVA) (SPSS 16.0 statistical software, SPSS Inc.). Pearson's correlation analysis was used to determine correlations among LMD, LVA, and LVL and the pterygium width, extension, and area. Values are presented as the mean ± standard deviation (SD). All reported values were 2-tailed, and differences were considered significant at *p* < 0.05.

## 3. Results

### 3.1. Immunohistochemical Staining

**Figure 1:**
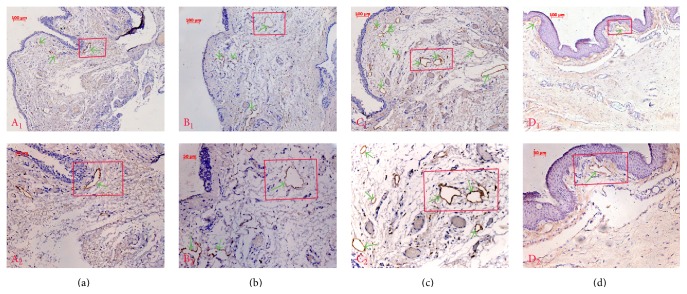
Lymphatic vessel endothelial hyaluronan receptor 1 (LYVE-1) immunohistochemistry for normal human conjunctiva and recurrent pterygia. A small number of LYVE-1 positive lymphatic vessels were found in Grade 1 (a) and 2 (b) recurrent pterygia. The number of lymphatic vessels was increased in Grade 3 (c) recurrent pterygia. The number of lymphatic vessels in normal human conjunctiva was decreased compared with that in pterygium patients. Only a few lymphatic vessels were positive for LYVE-1 in the normal epibulbar conjunctival segments (d) (green arrows indicate lymphatic vessels; magnification: A_1_, B_1_, C_1_, and D_1_  ×100; A_2_, B_2_, C_2_, and D_2_  ×200).

Lymphatic vessel development was examined by LYVE-1 immunohistochemistry in serial sections of human pterygia. LYVE-1, a hyaluronan receptor related to CD44, is a powerful marker of lymphatic structure and function and is expressed on the lymph vessel endothelial cells of both normal and neoplastic tissues and on both the luminal and abluminal surfaces of lymphatic endothelial cells [19, 20]. Our data demonstrated some LYVE-1 positive lymphatic vessels in the normal epibulbar conjunctiva segments. Lymphatic vessels were mildly increased in Grade 1 and 2 recurrent pterygia but were dramatically increased in Grade 3 recurrent pterygia (Figure 1).

### 3.2. Examination of the LVA, LMD, and LVL in Recurrent Pterygia

We examined the LVA, LMD, and LVL in Grade 1, 2, and 3 recurrent pterygia to elucidate the degree of lymphangiogenesis in patients with recurrent pterygia of differing severities. The LVA, LMD, and LVL were significantly increased in all three grades of recurrent pterygia compared with those in normal conjunctiva (*p* < 0.05). Although LVA, LMD, and LVL were slightly greater for Grade 2 pterygia than for Grade 1 pterygia, these differences were not significant (*p* > 0.05). LVA, LMD, and LVL were markedly elevated in Grade 3 pterygia, suggesting the dramatic development of conjunctival lymphangiogenesis (Table 2).

### 3.3. Relationship between Lymphangiogenesis and Recurrent Pterygium Width, Extension, and Area

**Figure 2:**
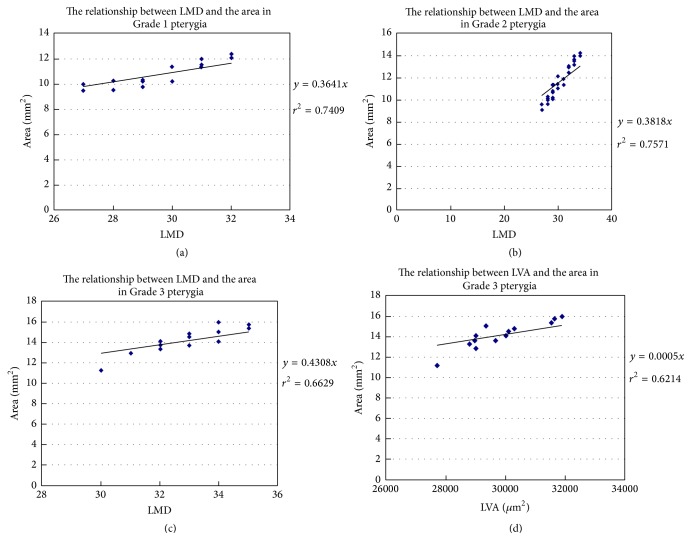
Relationships between lymphangiogenesis and the degree of aggression in recurrent pterygia. Significant relationships were found between LMD and pterygium area in Grade 1 pterygia ((a); *r* ^2^ = 0.7409, *p* < 0.05) and Grade 2 pterygia ((b); *r* ^2^ = 0.7571, *p* < 0.05). In Grade 3 pterygia, in addition to a significant relationship between LMD and pterygium area ((c); *r* ^2^ = 0.6629, *p* < 0.05), LVA was also significantly correlated with pterygium area ((d); *r* ^2^ = 0.6214, *p* < 0.05).

To elucidate the association of lymphangiogenesis with the degree of aggression in recurrent pterygia, we examined the relationships of the LVA, LMD, and LVL with the pterygium width, extension, and area in patients with the three grades of disease. A significant relationship was found between LMD and pterygium area in Grade 1 and Grade 2 pterygium patients (Figures 2(a) and 2(b)). The relationships between LVA and pterygium area and between LMD and pterygium area were significant in Grade 3 pterygium patients (Figures 2(c) and 2(d)). These findings suggested that increased severity of recurrent pterygia was associated with increased opportunities for conjunctival lymphangiogenesis.

## 4. Discussion

The identification of specific lymphatic endothelium markers has improved the understanding of the role of lymphangiogenesis in the pathogenesis of many diseases, including pterygium [21]. Blood vessels provide an entry route for immune effector cells (CD4^+^ alloreactive T lymphocytes and memory T lymphocytes), afferent lymphatic vessels enable the exit of antigenic material, and antigen-presenting cells (APCs) migrate to the regional lymph nodes and lymphoid organs [22–24]. Studies have suggested that lymphangiogenesis may be as important as angiogenesis in corneal immunity [25, 26]. Immunologic mechanisms likely contribute to the development of pterygia. Pterygium samples have increased levels of cell signaling and adhesion molecules such as vascular cellular adhesion molecule-1, intercellular adhesion molecule-1, E-cadherin, and b-catenin and aberrant expression of human leukocyte antigen-DR [27, 28]. Lymphangiogenesis in pterygia would provide new evidence of the involvement of immunological mechanisms in the growth of pterygia. Although angiogenesis has been extensively studied, lymphatic vessel development requires further research.

Cimpean et al. examined primary pterygium samples using D2-40 immunohistochemistry and reported an increased LMD in human pterygium compared with that in normal conjunctiva [29]. This report was the first to indicate active lymphangiogenesis in human pterygium. We found that both conjunctival blood and lymphatic vessels developed in primary pterygia; additionally lymphatic vessel outgrowth (lymphangiogenesis) may be more important than angiogenesis in more substantial pterygia [16]. However, Cimpean's and our previous studies only examined and calculated the MVD, including the LMD and BMD, in pterygia. The LMD does not adequately represent the changes of lymphatic vessels. Other parameters such as LVA and LVL are also needed to appropriately evaluate lymphangiogenesis status.

In one of our laboratory's prior studies, Ling et al. used computer software to simultaneously examine changes in LMD, LVA, and LVL and compared these changes for primary pterygia versus recurrent pterygia [30]. Their findings demonstrated that, in recurrent pterygia, there were increases in LVA (30%) that paralleled the observed increases in LMD. In addition, the expression of VEGF-C, the predominant lymphangiogenic factor, was more than the double in recurrent pterygia. Thus, lymphangiogenesis contributed to the formation of recurrent pterygia. Therefore, it was hypothesized that lymphatic vessels of patients with different pterygium severities must exhibit certain differences, perhaps with respect to quantity. However, Ling et al. only discussed lymphangiogenesis differences between primary and recurrent pterygia and did not carry out comparison with lymphatic vessels for different pterygium severities. More evidence is necessary to clarify whether changes in lymphatic vessels are correlated with the degree of aggression observed in recurrent pterygia.

In the current study, we divided pterygium patients into 3 groups based on clinical evaluations performed using the methods described by Awdeh et al. [18]. We examined pterygium extension, width, and total area to assess the degree of pterygium aggression in each group. Total pterygium area was significantly greater among Grade 2 patients than among Grade 1 patients. Although pterygium extension and width were slightly greater in Grade 2 patients than in Grade 1 patients, these differences were not significant (*p* > 0.05). Pterygium extension, width, and area were markedly greater for Group 3 patients than for Grade 1 and Grade 2 patients (Table 1). Thus, the degree of aggression of recurrent pterygia was most severe, moderate, and mild in Grade 3, Grade 2, and Grade 1 cases, respectively. We examined how LVA, LMD, and LVL related to pterygium extension, width, and area in case of various grades. One relationship (between LMD and the pterygium area) was significant for Grades 1 and 2. Two relationships (between LVA and the pterygium area and between the LMD and the pterygium area) were significant for Grade 3. Therefore, lymphatic vessel outgrowth may be important in substantial recurrent pterygia (Grade 3).

The relationship between the LVL and the degree of pterygium aggression was not significant. Although the LVL was significantly increased in recurrent pterygium patients compared with that in the controls, the LVL was not correlated with the pterygium width, extension, or area, even in Grade 3 patients, who exhibited increased LVL. Conjunctival lymphatic vessels, similar to the corneal limbus lymphatic arcade, may lack the classical valve structure and belong to lymphatic capillaries. However, the LVL of conjunctival lymphatic capillaries was larger than that of the initial lymphangiogenic capillaries. The initial lymphangiogenic capillaries may influence pterygium aggression more than the existing conjunctival lymphatic capillaries. This result may explain the lack of a significant relationship between the LVL and the degree of pterygia.

## 5. Conclusions

In conclusion, significant increases in LVA, LMD, and LVL were found in recurrent pterygia. In addition, conjunctival lymphangiogenesis was closely associated with pterygium development and formation, particularly substantial Grade 3 pterygia. Lymphatic vessels may accelerate immunological injury and play a key role in the immunopathological mechanisms that result in pterygia. Antilymphangiogenic therapy may improve the efficacy of pterygium interventions and the prognoses of these patients.

## Figures and Tables

**Table 1:** Composition of recurrent pterygia.

Pterygia patients	Pterygia grade		
	Grade 1 (*n* = 19)	Grade 2 (*n* = 28)	Grade 3 (*n* = 13)
Pterygium width (mm)	4.02 ± 0.75	4.30 ± 0.86^#^	6.18 ± 1.03^*∗*+^
Pterygium extension (mm)	3.20 ± 0.31	3.22 ± 0.32^#^	3.53 ± 0.29^*∗*+^
Pterygium area (mm^2^)	10.68 ± 0.88	11.46 ± 1.54^*∗*^	14.16 ± 1.27^*∗*+^

Data are expressed as means ± SD; *∗* and #, *p* < 0.05 and *p* > 0.05, respectively, compared with Grade 1; ^+^ *p* < 0.05 compared with Grade 2.

**Table 2:** Comparative evaluation of lymphatic vessels in recurrent pterygia.

Patients	Number	LVA (*μ*m^2^)	LMD (*n*)	LVL (*μ*m)
Grade 1 pterygia	19	27608.2 ± 684.3^&#+^	29.4 ± 1.5^&#+^	174.3 ± 5.5^&#+^
Grade 2 pterygia	28	28188.4 ± 1202.5^&+^	30.1 ± 2.2^&+^	177.0 ± 6.4^&+^
Grade 3 pterygia	13	29845 ± 1244.9^&^	32.9 ± 1.5^&^	185.3 ± 4.4^&^
Controls	9	12181.1 ± 226.3	10.2 ± 1.0	128.8 ± 8.1

Data are expressed as means ± SD; ^#^ *p* > 0.05 compared with Grade 2; ^+^ *p* < 0.05 compared with Grade 3; ^&^ *p* < 0.05 compared with controls.
